# Beat-to-Beat Blood Pressure and Two-dimensional (axial and radial) Motion of the Carotid Artery Wall: Physiological Evaluation of Arterial Stiffness

**DOI:** 10.1038/srep42254

**Published:** 2017-02-13

**Authors:** Chenchu Xu, Huahua Xiong, Zhifan Gao, Xin Liu, Heye Zhang, Yanping Zhang, Xiuquan Du, Wanqing Wu, Guotao Liu, Shuo Li

**Affiliations:** 1 School of computer Science and Technology, Anhui University, Hefei, 230601, China; 2 Department of Ultrasound, Shenzhen Second People’s Hospital, Shenzhen, 518000, China; 3 Shenzhen Institutes of Advanced Technology, Chinese Academy of Sciences, Shenzhen, 518055, China; 4 Shenzhen College of Advanced Technology, University of Chinese Academy of Sciences, Shenzhen, 518055, China; 5 Department of Medical Imaging, Schulich School of Medicine and Dentistry, University of Western Ontario, 1151 Richmond St, London ON, Canada

## Abstract

The physiological relationship between local arterial displacement and blood pressure (BP) plays an integral role in assess- ment of the mechanical properties of arteries. In this study, we used more advanced methods to obtain reliable continuous BP and the displacement of the common carotid artery (CCA) simultaneously. We propose a novel evaluation method for arterial stiffness that relies on determining the physiological relationship between the axial and radial displacements of the CCA wall and beat-to-beat BP. Patients (total of 138) were divided into groups according to the following three criteria: essential hyper- tension (EH) and normotension, male and female, elderly and younger. The Pearson correlation test and canonical correlation analysis showed that the CCA indices were significantly correlated with BP indices (*r* = 0:787; *p* < 0:05). The slope of the CCA displacement/pressure curve showed a progressive reduction with increasing age and EH disease occurrence (EH: 0.496 vs. normotension: 0.822; *age* <= 60:0.585 vs. *age* > 60:0.783). Our method provides an explicit reference value and relationship for the manner in which the CCA wall responds to changes in BP. Short-term and continuous BP were significantly correlated with CCA displacement and exhibited a close inverse relationship with each subject’s BP and EH, age, and systolic blood pressure.

Atherosclerosis has been considered the main cause of morbidity and mortality in relation to cardiovascular diseases. As a result, the evaluation of arterial stiffness has become a popular research field, receiving wide attention^1^. In previous studies, substantial evidence has demonstrated that arterial stiffness undergoes a progressive change with aging, yielding values that are markedly changed in cases of hypertension and a variety of other diseases^2^,^3^,^4^. Such changes are also known to give rise to adverse clinical consequences. Arterial wall displacement refers to the deformation and rate of deformation of an arterial wall in axial, planes, and circumferential planes. Increased stiffness of the large central arteries, as estimated by the common carotid artery (CCA) wall displacement, is a risk factor for atherosclerosis^5^,^6^,^7^. However, blood pressure (BP) is the pressure exerted by the circulating blood on the walls of blood vessels. The displacement of the arterial wall is associated with the development of stresses due to the BP and blood flow, as well as tethering into the surrounding tissues^8^. Through observing a temporal relationship between the blood velocity wave and anterograde carotid artery longitudinal wall displacement, the relationship between carotid artery movement and left ventricular rotation has been found^9^. Several researchers have revealed the relationship between blood pressure variability (BPV) and alterations to the artery, identifying the mean level of and variability in the BP as potential risk factors for arterial stiffness^10^,^11^. Therefore, the accurate evaluation of the physiological relationship between arterial wall displacement and changes in pulse pressure is important for exploring the mechanism of arterial stiffness, which has an important clinical value.

The displacement of the arterial wall has been under study for many decades. As a physical phenomenon, the radial displacement of the artery is easy to explain based on the combined action of variations in the BP gradient, the elastic properties of the arterial wall, and the periphery of the pressure wave reflection^12^. The axial expansion of the arterial wall is a relatively new phenomenon. It was first discovered in the 1950s, although it is typically considered to be negligible compared to the radial expansion of these vessels^13^. Due to medical imaging technical problems with participant observation of the distension of the axial artery wall, the axial displacement of the arterial wall remains a more complex phenomenon that is harder to measure than radial displacement. Recently, improvements in ultrasound resolution have contributed to the speckle tracking image analysis method that has been used to quantify arterial wall movements in two dimensions. Speckle tracking uses a block matching scheme to track the position of speckle patterns in carotid artery tissue through a sequence of b-mode ultrasound images^14^. At present, many research achievements based on speckle tracking ultrasound imaging to estimate CCA wall mechanics have been obtained^15^,^16^,^17^,^18^. For instance, the association between the acute double-leg press exercise and similar transient changes in CCA wall mechanics at low and moderate intensities has been demonstrated^19^. Similarly, the BPV is an important factor in assessments of end-organ damage, independent of the traditional BP level; the target organs of such studies have mainly been the heart, brain, and kidneys^20^. With a high BPV, CCA wall remodeling is mainly characterized by an increase in the intima media thickness (IMT), which allows the vessel to maintain its circumferential wall stress but changes the stiffness and thickness of the CCA. CCA short-axis ultrasound images have shown that circumferential wall displacement of the CCA wall is significantly related to pulse pressure^21^. Moreover, increased pulse pressure as well as increased stiffness and thickness of the CCA wall are known to be significant, independent predictors of cardiovascular complications^22^. Changes in the stiffness and thickness of the CCA are determinative effects for displacing the CCA wall. However, in previous studies, most BPV parameters were captured either from 24-hour ambulatory BP monitoring (ABPM) or following several BP measurements recorded during home visits using clinical BPV^23^,^24^,^25^. As a result, many current methods: (1) focus solely on associations between carotid artery longitudinal displacements and cardiovascular disease risk factors^26^; and (2) measure continuous BP, which is not accurate and is estimated by complex mathematical assumptions^27^. (3) Simultaneously obtaining reliable short-term BP measurements and measurements of axial and radial (2-D) displacement of the CCA wall at same time period has not been investigated.

The aim of this study was to determine the relationship between two-dimensional (axial and radial) motions of the CCA wall and beat-to-beat BP to explore the mechanism of arterial stiffness in longitudinal ultrasound images. We aimed to solve the limitations of current methods for assessing carotid artery motion properties in which continuous BP is estimated by complex mathematical assumptions and arterial expansion evaluations are not accurate enough. Our method may have several advantages: (1) an index of arterial dispensability that includes the integrated characteristics and interactive influence of axial and radial displacements are provided, which ensures the influence of each individual indicator; (2) BP is used as a new, continuous physiological parameter and the issue of how 2-D arterial function varies on a beat-to-beat BP basis has never been addressed previously; (3) A more accurate 2-D displacement of the CCA has been obtained by a novel and reliable motion-tracking algorithm; and (4) it simultaneously obtains a reliable short-term BP measurement and measurement of 2-D displacement of the CCA wall at same time.

## Materials and Methods

### Study Participants

The study protocol was designed according to the principles of the Declaration of Helsinki and was approved by the Ethics Committee of the Second People’s Hospital of Shenzhen in China. The study objectives and design have not been previously published. All participants provided written informed consent. The study population consisted of 138 Chinese participants aged 29 to 81 years (59.6 ± 11.4 years). Among the participants, 85 participants were suffering from essential hypertension and 19 participants were diagnosed with diabetes. Risk factors such as smoking, alcohol, and body mass index were recorded.

### Beat-to-beat BP and Carotid Arterial Displacement

**Figure 1:**
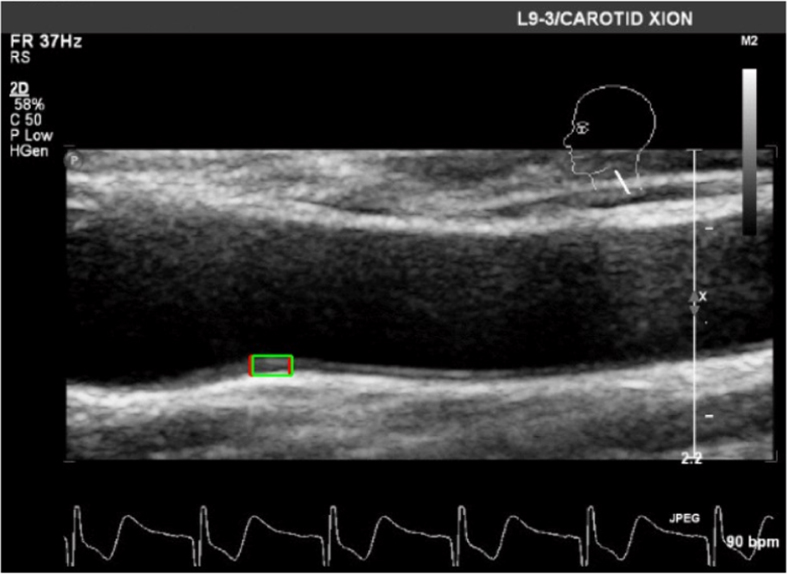
Image of the carotid artery wall displacement estimated from the b-mode ultrasound using region tracking and block matching.

In the experiment, both the beat-to-beat BPs and CCA displacements were gathered as continuous data, Hence to complete our analysis, we needed to measure the values at the same time. Beat-to-beat recordings of the systolic BP (mmHg) and diastolic BP (mmHg) were measured using a Finometer system Model-2 (Midi). A cuff was attached to the left upper arm close to the elbow for continuous beat-by-beat measurement of BP. For both the entire group and each of the individuals in this study, one ultrasound physician with more than 10 years of experience measured and evaluated the axial and radial displacements of the left and right carotid artery walls using a high-resolution ultrasound system (iU22, Philips Ultrasound, Bothell, WA, USA) and 7.5 MHz linear array transducer. During collection, all of the measurements were made with patients in a supine position. Scanning was performed on both CCAs in the lateral plane, 2–5 cm proximal to the carotid bifurcation, so that the CCA segment was displayed as a rectilinear structure (Fig. 1). Video clips of six consecutive cardiac cycles were obtained for each participant. All imaging data were saved in both the DICOM format and as CDs for off-line analysis.

### Tracking the 2D displacement of the Carotid Artery Wall

The 2D displacement of the carotid artery wall in the ultrasound sequence was tracked using the H_∞_-based block matching method (HBM). The HBM uses the state-space equation to describe the motion of the target tissue on the carotid artery wall as below^28^


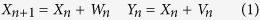


where *X*_*n*_ is the estimated location of the target tissue, *Y*_*n*_ is the observed location of the target tissue acquired by the block matching method, *W*_*n*_ and *V*_*n*_ are noise terms, and *n* is the time or frame index. Then, the H_∞_ filter was used to compute the optimal estimate of the target tissue location using a min-max approach. Given the worst possible disturbances from the data, our HBM method can still ensure optimal estimation of carotid artery wall motion^29^.

### Carotid Artery and Blood Pressure Curves

**Figure 2:**
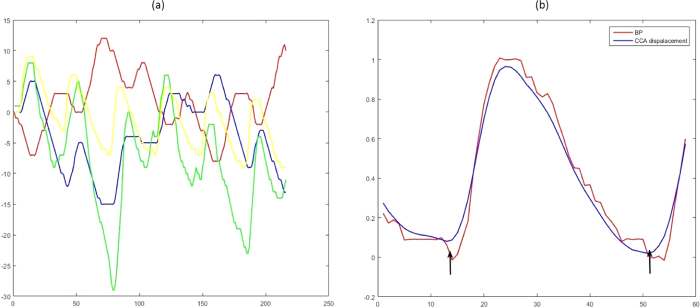
(**a**) Axial and radial displacement of the left and right carotid artery walls. The red line represents the axial displacement of the left CCA. The blue line represents the radial displacement of the left CCA. The green line represents the axial displacement of the right CCA. The yellow line represents the radial displacement of the right CCA. (**b**) An initial alignment of the pressure and CCA displacement waveforms.

According to the separate axial and radial displacements of the left and right carotid artery walls and BP signals, 4-type displacement/pressure curves were obtained via software, which was developed with MATLAB. From the recording time, an initial alignment of pressure and 2-D displacement waveforms was obtained. Then, a correction factor of 30–60 milliseconds was applied to the CCA displacement to correct the delay due to the propagation of the carotid artery pressure wave. It was also applied for removal of nonlinear waveforms and dealing with semiautomatic irregular waveforms. Once aligned, the beginning and end of the segments to be analyzed within each waveform were visually identified on a complete cardiac cycle between two late diastole phases (Fig. 2). When CCA displacement/pressure curves were calculated, the curve fitting method was used to estimate the best slope for the curve.

### Carotid Artery and Blood Pressure Assessments

The motion of the CCA wall includes axial motion in the left CCA wall (LAM), radial motion in the left CCA wall (LRM), axial motion in the right CCA wall (RAM), and radial motion in the right CCA wall (RRM). The systolic blood pressure variability (SBPV) and diastolic blood pressure variability (DBPV) were examined using the systolic blood pressure (SBP) and diastolic blood pressure (DBP). The measurements from the beat-to-beat BP and motion of the CCA wall were assessed by the mean level and variability indices in the time and frequency domains. The BP indices in the time domain included the mean value (MEAN), standard deviation (SD), coefficient of variation (CV), successive variation (SV), average real variability (ARV), and residual standard deviation (RSD)^24^,^30^,^31^,^32^. The CCA indices, as with the BP indices, could be computed to reflect the characteristics of the four types of CCA wall motion. The definitions of these indices are listed in Table 1.

### Canonical Correlation Analysis

Canonical correlation analysis is an approach for making sense of cross-covariance matrices. When there are two vectors *X* = (*x*_1_, …, *x*_*n*_) and *Y* = (*y*_1_, …, *y*_*m*_) of random variables, and there are correlations among the variables, canonical correlation analysis will identify linear combinations of *X*_*i*_ and *Y*_*i*_ that are maximally correlated with each other^33^:


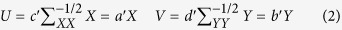


where *U* and *V* are the first pair of canonical variables. The vectors *a* and *b* such that the random variables *a*′*X* and *b*′*X* maximize the correlation *ρ* = *corr(a*′*X, b*′*Y*). Canonical correlation analysis is the general procedure for investigating the relationships between two sets of variables^34^.

Canonic correlation analysis is used to investigate the relationships between two sets of variables and it is preceded by a hypothesis test. In this case, we used Lilliefors test to validate our data satisfying the normal distribution (lillietest, MATLAB). The Pearson’s linear correlation coefficient was used to detect multiple co-linear relationships among the variables. The results showed that there was a correlation between the variables, but not a strong correlation (r = −0.323–0.46, p < 0.05). Meanwhile, multivariate statistics and F approximations demonstrated that our data satisfied the linear correlation between the variables group and canonical correlation coefficients significantly (Wilks’lambada and F value, p value).

### Statistical Analysis

Pearson’s linear correlation coefficient test was employed to measure the correlation between the BP and CCA indices. The Benjamini & Hochberg method was used to construct multiple comparison corrected confidence intervals that corresponded to the FDR-adjusted p-values^35^. Then, the canonical correlation analysis was used to assess the interrelation and interactions of the CCA indices and BP indices. Finally, according to whether participants had essential hypertension and their sex and age, we grouped all of the participants and identified the similarities and differences between each group. The results of this analysis are demonstrated in the carotid artery displacement/blood pressure curves and canonical correlation analysis. We implemented all codes using MATLAB R2012a on a desktop computer with Intel(R) Xeon(R) CPU E5-2650 (2.00 GHz) and 32 GB DDR2 memory. The statistical analysis was implemented with SPSS (Statistical Product and Service Solutions) 23.0 using a statistical software package (IBM, Armonk, NY), and a calculated difference of P < 0.05 was considered statistically significant.

## Results

### Baseline Characteristics

Participants were a mean (standard deviation) of 59.6 (11.4) years old, and 76 (55.1%) were male. The participants included two diseases: 19 (13.8%) had diabetes, and 76 (55.1%) had essential hypertension. The smoking, alcohol and coffee rates were 23.2%(32), 14.1%(20) and 2.8%(4), respectively. The mean SBP was 140.2 (23.3) mmHg, and the average DBP was 74.5 (13.4) mmHg. The mean LAM was −0.8 (28.6) pixels, and the mean LRM was 3.7 (9.9) pixels. The mean RAM was −3.8 (25.7) pixels, and the mean RRM was 3.6 (9.3) pixels.

### Beat-to-beat BP Index Associations with Left CCA Indices

Tables 2 and 3 show the Pearson’s linear correlation coefficients between CCA indices and BP indices after adjustment of p-values by Benjamini & Hochberg method. A total of 288 sets of correlation coefficients were calculated; 51 sets were significantly correlated with each other (*r* = −0.323–0.462, *p* < 0.05). In particular, the residual standard deviation of axial displacement in the left CCA wall (*LAM*_*SV*) was the strongest positive that was significantly correlated with the coefficient of variation of the systolic blood pressure (*SBPV*_*SD*) (*r* = 0.105, *p* < 0.05), and *LRM*_*RSD* was the strongest positive that was significantly correlated with the *SBPV*_*RSD (r* = 0.462, *P* < 0.05). *LRM*_*SV* was the strongest negative that was significantly correlated with the *DBPV*_*MEAN (r* = −0.242, *p* < 0.05), and *LAM*_*ARV* was the strongest negative that was significantly correlated with the *DBPV*_*MEAN (r* = −0.323, *p* < 0.05).

### Canonical Correlation Analysis

Canonical correlation analysis was used to test the integration relativity between the CCA indices and BPV indices. It was also used to study the positive and negative factors in the correlation.There was a strong canonical correlation between the CCA and BP indices (*r* = 0.787, *p* < 0.05). Furthermore, the *SBPV*_*SD, DBPV*_*SD, DBPV*_*CV DBPV*_*SV, LRM*_*SD*, and *LRM*_*ARV* were the major positive factor relative contributions to the relationship. Similarly, the *SBPV*_*CV, SBPV*_*RSD, DBPV*_*RSD, LRM*_*RSD*, and *RRM*_*ARV* were the major negative factor relative contributions to the relationship. Table 4 presents the canonical correlation analysis between beat-to-beat BP indices and CCA indices.

### Essential Hypertension Disease Subgroup Analyses

**Figure 3:**
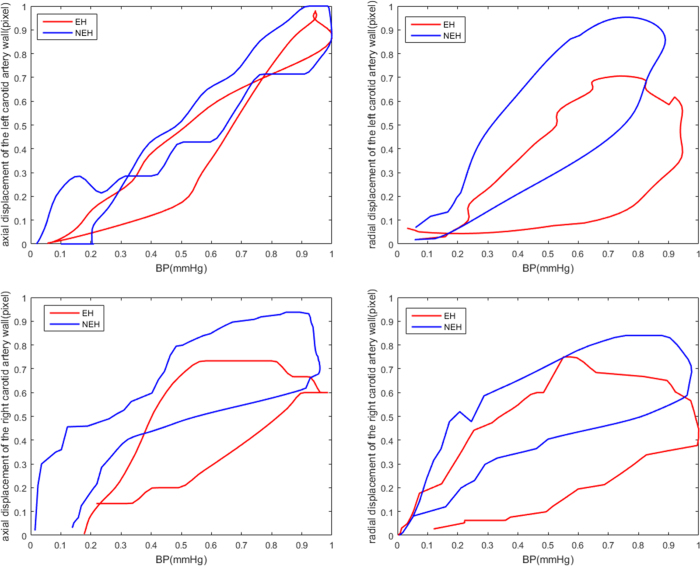
2-D displacement of the left and right carotid artery and pressure signals and their relationship with EH and NEH throughout the cardiac cycle.

According to whether participants had essential hypertension (EH), all of the subject were subdivided into the EH and NEH groups. As shown in Fig. 3, both in the axial and radial dimensions, the slope of the CCA displacement/pressure curve was lower in EH than in NEH participants (*EH*:0.496*vs.NEH*:0.822). Canonical correlation analysis was also used to measure the relativity between the CCA indices and BP indices in both groups, revealing major differences in the contributing factors. There were significant correlations between the CCA and BP indices for both groups (*r* = 0.907*andr* = 0.919, *respectively*; *p* < 0.05). The major positive influencing factors in the EH group were *SBPV*_*CV, SBPV*_*SV, DBPV*_*SD, LRM*_*SD*, and *LRM*_*ARV*, and the major negative influencing factors were *SBP*_*SD, SBP*_*ARV*, and *LRM*_*RSD*. The major positive influencing factors in the NEH group were *SBPV*_*SD, DBPV*_*CV, LRM*_*SD*, and *LRM*_*ARV*. The major negative influencing factors are *SBPV*_*CV, SBPV*_*RSD, DBPV*_*RSD*, and *LRM*_*RSD*. These findings are summarized in Table 5.

### Sex Subgroup Analyses

**Figure 4:**
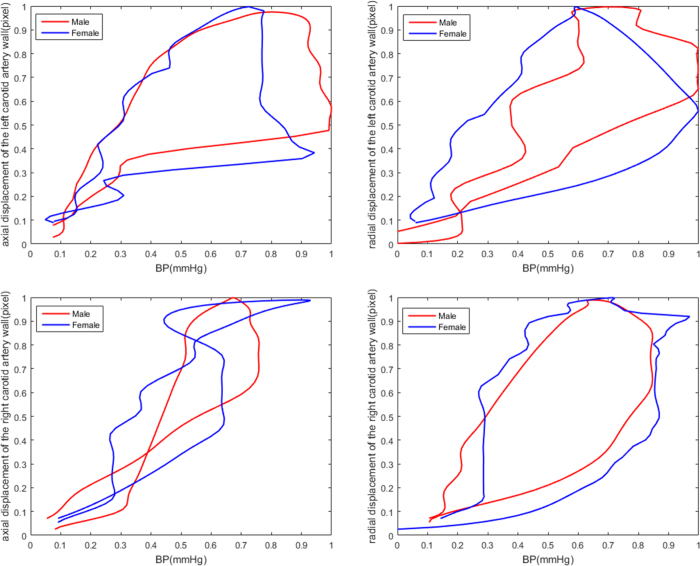
2-D displacement of the left and right carotid artery and pressure signals as well as their relationship with female and male sex throughout the cardiac cycle.

Figure 4 and Table 6 present the CCA displacement/pressure curve and different correlations between CCA and BP indices as well as the interrelation between each influencing factor in males and females. The average slope of the male curve was similar to that in females (0.861*vs*.0.808), and there were significant correlations between CCA and BP indices for both groups (*r* = 0.940*andr* = 0.919, *respectively*; *p* < 0.05). However, the *SBPV*_*CV, SBPV*_*SV, DBPV*_*ARV*, and *DBPV*_*RSD* mostly had positive coefficients and the *SBPV*_*SD, SBPV*_*ARV, SBPV*_*RSD, DBPV*_*SV*, and *LAM*_*ARV* had significantly negative coefficients for the correlation in the male group. Similar results were recorded in the female group.

### Age Subgroup Analyses

**Figure 5:**
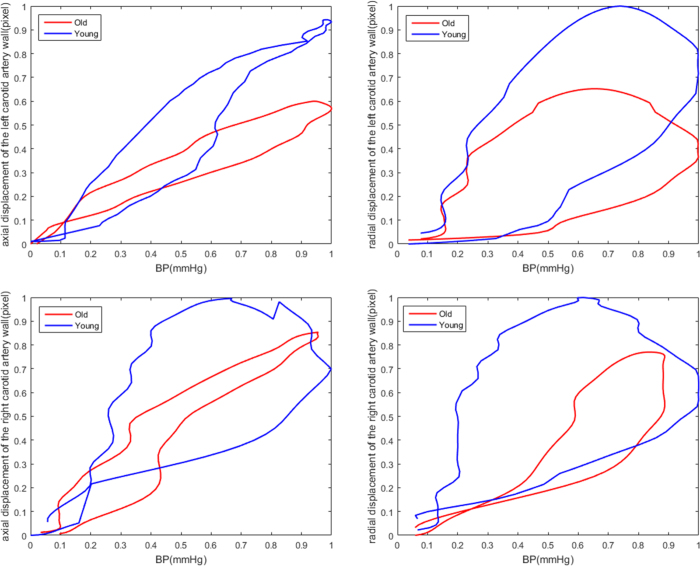
2-D displacement of the left and right carotid artery and pressure signals as well as their relationship with older and younger ages throughout the cardiac cycle.

**Figure 6:**
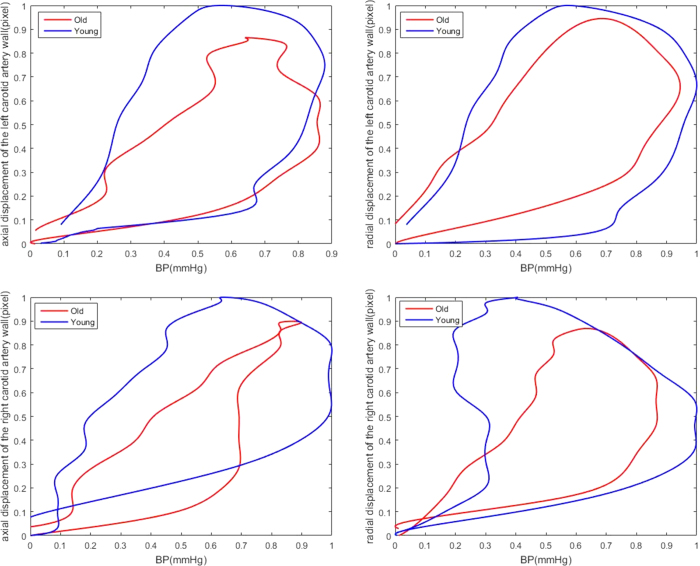
2-D displacement of the left and right carotid artery and pressure signals as well as their relationship with older and younger ages throughout the cardiac cycle (without EH).

Participants in the elderly group (*age* > 60) had lower slopes compared to the younger group (*age* <= 60), as shown in Figs 5 and 6 (including EH: 0.585*vs*.0.783; without EH: 0.572*vs*.0.667, respectively). Similar to the results described above, Tables 7 and 8, show both groups yielded significant canonical correlations between the CCA and BP indices (including EH: *r* = 0.870 and 0.926; without EH: *r* = 0.890 and 0.882, respectively; *p* < 0.05). *DBPV*_*SD, DBPV*_*CV, DBPV*_*CV, DBPV*_*SV*, and *RAM*_*SD* were significantly positively correlated in the elderly group. *SBPV*_*CV, SBPV*_*RSD, DBPV*_*ARV, DBPV*_*RSD*, and *LRM*_*RSD* had a significant positive effect on the correlation. In contrast, *SBPV*_*CV, SBPV*_*RSD, DBPV*_*RSD, LAM*_*SD, LAM*_*SD*, and *RRM*_*SD* were major positive correlation factors, and *SBPV*_*SD, SBPV*_*ARV, DBPV*_*SD, DBPV*_*CV, DBPV*_*SV, LRM*_*SD, LRM*_*ARV*, and *RRM*_*RSD* were major negative correlation factors in the younger group.

## Discussion

In this study, we estimated the 2-D displacement of the carotid artery wall and beat-to-beat BP as well as the influence of age, sex, and essential hypertension on the estimations. Our goal was to conduct preliminary trials that validated the interrelation and interaction between the CCA and BP indices as well as to assess the mechanical properties of the artery. In previous studies, a series of effective methods for studying the axial and radial movements of the arterial wall was presented^36^,^37^,^38^,^39^. Our method was as follows: (1) We used a H_∞_ filter based standard block matching method (BM) method to estimate the motion of the carotid artery wall from the ultrasound image sequences^28^. This approach used a newer software version with a free trace feature that did not make any assumptions about the vessel geometry and allowed for more precise measurements of both the axial and radial displacements of the left and right carotid artery walls; (2) Compared with the traditional methods of ABPM or readings from several BP measurements in home visits using clinical BPV, beat-to-beat recordings of BP offered a new, continuous physiological parameter. This approach is recommended as the best means to capture short-term BPV, while intermittent ABPM is less precise^24^. (3) Precise matching of the changes in artery 2-D displacement was carried out with the actual changes in intravascular pressure occurring at the same time. It helped us make more precise observations of how the CCA wall responded to blood pressure changes; and (4) Canonical correlation analysis was the best way to produce a model equation that related two sets of variables. The analysis relied on slopes to express both the displacement and pressure as continuous data.

The CCA wall invariably undergoes displacement with blood pressure changes because the essence of BP is the pressure exerted by circulating blood on the blood vessel walls. In our experiments, the most important finding was a strong canonical correlation between the beat-to-beat BP and carotid artery wall displacement, indicating that the two-dimensional motion of the carotid artery wall has a strong influence on the beat-to-beat BPV. This result is consistent with the findings presented in a previous publication, namely, that a basic physiological feature, the variability in the BP level, is a significant marker of the risk related to cardiovascular complications^32^,^40^,^41^. BP is a strong determinant of cardiovascular events, and aortic stiffening is highly associated with the progression to high BP in humans. Arterial stiffening plays a pivotal role in the progression of high BP and the development of cardiovascular disease^23^. Arterial stiffness occurs as a result of arteriosclerosis, which results from long-term cumulative damage in the artery^42^. Our experimental results provided a precise assessment function, which can allow for the identification, monitoring, and characterization of arteriosclerosis patients in non- or pre-clinical studies. Simultaneously, the impact of both positive and negative influencing factors on the result was clearly explained. We evaluated the relationship between the response of the CCA wall and the changes of the BP within the cardiac cycle in 138 participants as well as in multiple mechanical properties of the elastic artery.

Age, sex, and disease status, and possibly other independent risk factors, were also strongly and inversely associated with carotid displacement or BPV. Svedlund and Gan suggested that differences in age might have confounded the results of prior studies that showed impaired carotid longitudinal displacement in older adults with coronary artery disease and type- 2 diabetes^43^. Zhang *et al*. observed that the buffer effect of blood vessels can be enhanced by systemic structural changes in the arteries with age, which leads to increase SBP and, potentially, increased pulse pressure^44^. Our trial suggests that there is a highly significant correlation between carotid artery wall movement and BPV, whether participants are younger or older, male or female, or diagnosed with essential hypertension or not, which is consistent with our above result. The slope of the CCA displacement/pressure curve showed a progressive reduction with increasing age and EH disease status. It is a well-known phenomenon that arterial stiffness results in arterial displacement changes, the arterial stiffness rate increases with increasing age, and EH is one of the main diseases that affects arterial stiffness. While several clinical investigations have examined the association between menopause and the atherosclerotic cardiovascular risk, estrogen deficiency can protect females and mediate arterial stiffening with aging^45^,^46^. However, in our study, the male and female groups had nearly identical slopes of the CCA displacement/pressure curves. We can speculate that this similarity is because the average age of participants was high and most participating females were postmenopausal. Additionally, there were small differences between the axial and radial displacements and the left and right CCA in consecutive cardiac cycles, suggesting that modulation of the arterial mechanical response to continuous changes in intravascular pressure may undergo physiological variations.

ARV is a more appropriate parameter to measure variability, which provides a computationally simple way to estimate variation in a trend. As for ARV, RSD tends to be larger in absolute value and is influenced, to a greater extent, by large discrepancies between successive measurements. In our Pearson’s linear correlation analysis, the ARV and RSD of carotid artery wall displacement were more significantly correlated with BP indices. We also found the ARV and RSD of carotid artery wall movement had the same tendency of changes with the same BP indices in standardized canonical correlation coefficients. It suggested that ARV and RSD are the most appropriate predictors when expressing the relationship between CCA wall displacement and BP.

The common carotid artery is present on the left and right sides of the body. The left common carotid artery can be considered to have two parts, the thoracic (chest) and cervical (neck) regions. The right common carotid originates in or close to the neck, containing only a small thoracic portion^47^. Only the left common carotid artery has a substantial presence in the thorax. It originates directly from the aortic arch and travels upward through the superior mediastinum to the level of the left sternoclavicular joint. In canonical correlation analysis, the mechanical properties of the common carotid arteries support our findings and suggest that the *LRM*_*SD* and *LRM*_*ARV* are related to the *SBPV*_*SD, DBPV*_*SD, DBPV*_*CV*, and *DBPV*_*SV*. The data for the left carotid artery were more representative of the influence of the BP on the CCA wall than the data for the right carotid artery. Additionally, we also found the CCA radial displacements were more related to the BP indices than axial displacements, indicating that the changes in BP had more influence on CCA radial than axial displacements. BP plays a crucial role in vascular biology, and may affect IMT through blood vessel remodeling or wall hypertrophy in response to altered circumferential stress^48^. Meanwhile, IMT is a complex process, leading to changes in the local hemodynamics, shear stress and circumferential tensile stress. Those changes may cause the above mentioned results and thus possibly interferes with the axial displacement of the intima-media.

Fully elucidating the mutual roles of CCA wall displacement and the changes in BP based on our limited research is a difficult task as these elements appear to act in a complex system, impacting each other. However, some practical significance results can be obtained by our canonical correlation coefficients. For example, we found that a smaller *SBPV*_*CV* means a smaller *LRM*_*RSD* based on overall canonical correlation analysis. This enhanced the study in regard to traumatic effects of intravascular pressure excursions on the vessel wall, thereby favoring atherosclerosis formation mechanism research. Similarly, our 2-D artery and BP curves have also verified the slopes of the 2-D displacement-pressure relationship can be obtained from the carotid arteries during most of the ascending and descending portion of the pulse pressure wave, which may allow study of the mechanisms of this CCA variability and their possible alterations with disease. Additionally, the study of EH can be used to understand more about changing BP and stress levels and short-term changes in endothelial function and how they lead to some degree of variation in the reproducibility results.

There are certain limitations in our study. First, we chose a certain set of characteristics for evaluation and we grouped the participants without considering other known and confounding factors (smoking, alcohol and caffeine status) in the study population. Similarly, cultural factors, ethnic differences, and factors such as socioeconomic disadvantages and psychosocial stress may have contributed to the observed differences between the groups. Second, we focused on determining the relationship between the axial and radial displacements of the CCA wall and BP based on longitudinal ultrasound images. Therefore, more research should consider the influence of the circumferential displacement of the CCA wall to our results in the next stage, which also plays an important role in arterial wall mechanics and measurements based on short-axis ultrasound images. Similarly, simultaneous measurement avoids the error that occurs when measurements are made asynchronously. However, we must acknowledge the limitations that our key parameters were measured in different arteries; the blood pressure was measured in the brachial artery and arteria wall displacement was measured in the carotid artery. This limitation should also be investigated further. Finally, further research is needed on the relative contributions of both positive and negative factors for the CCA and beat-to-beat BP indices to improve our understanding of the relationships.

## Conclusions

The two-dimensional motion of the carotid artery wall had a significant correlation with beat-to-beat BP indices, and positive or negative factors that contributed to the relationship were clearly identified. Additionally, we observed that the variation in the carotid artery wall displacement and BPV features could be influenced by multiple risk factors, including the age, sex, and essential hypertension status. Moreover, this new method allows for a precise, in-depth assessment of the carotid artery mechanical properties at rest. However, more research is required to validate these correlations under physiological stress.

## Additional Information

**How to cite this article**: Xu, C. *et al*. Beat-to-Beat blood pressure and Two-dimensional (axial and radial) motion of the Carotid Artery Wall: physiological evaluation of arterial stiffness. *Sci. Rep.* **7**, 42254; doi: 10.1038/srep42254 (2017).

**Publisher's note:** Springer Nature remains neutral with regard to jurisdictional claims in published maps and institutional affiliations.

## Figures and Tables

**Table 1:** Measures of variability for a set of n measurements x1, x2, x3.

Measure	Definition	Equation
MEAN		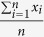
SD	Standard deviation	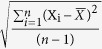
CV	Coefficient of variation	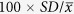
ARV	Average real variability	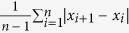
SV	Successive variation	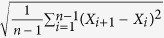
RSD	Residual standard deviation	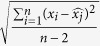

SD standard deviation, CV coefficient of variation, ARV average real variability, SV successive variation, and RSD residual standard deviation.

**Table 2:** Correlation between beat-to-beat BP and left CCA indices.

	LAM_MEAN		LAM_SD		LAM_CV		LAM_ARV		LAM_SV		LAM_RSD	
	r	P	r	P	r	P	r	P	r	P	r	P
SBPV_MEAN	−0.042	0.928	0.027	0.928	−0.014	0.928	−0.091	0.763	−0.064	0.928	0.012	0.928
SBPV_SD	0.1	0.342	0.166	0.101	−0.001	0.995	0.223*	0.024	0.105*	0.006	0.163	0.101
SBPV_CV	0.118	0.224	0.162	0.099	0.008	0.93	0.266*	0.012	0.123	0.21	0.168	0.09
SBPV_ARV	0.06	0.594	0.119	0.305	−0.029	0.737	0.229*	0.019	0.15	0.17	0.105	0.368
SBPV_SV	0.087	0.482	0.071	0.543	−0.053	0.615	0.163	0.112	0.085	0.482	0.064	0.576
SBPV_RSD	0.096	0.351	0.188	0.054	−0.021	0.839	0.225*	0.024	0.114	0.277	0.183	0.059
DBPV_MEAN	0.011	0.898	−0.117	0.342	0.056	0.616	−0.323*	0	−0.275*	0.012	−0.157	0.203
DBPV_SD	0.062	0.735	0.12	0.386	−0.024	0.786	0.109	0.404	0.039	0.786	0.098	0.453
DBPV_CV	0.083	0.472	0.163	0.137	−0.038	0.749	0.243*	0.016	0.147	0.147	0.152	0.147
DBPV_ARV	0.091	0.686	0.025	0.913	−0.017	0.915	0.085	0.704	0.07	0.708	−0.013	0.916
DBPV_SV	0.083	0.664	0.002	0.989	−0.035	0.966	0.054	0.867	0.03	0.967	−0.021	0.972
DBPV_RSD	0.061	0.692	0.134	0.314	−0.037	0.76	0.099	0.429	0.04	0.76	0.108	0.386
	**LRM_MEAN**		**LRM_SD**		**LRM_CV**		**LRM_ARV**		**LRM_SV**		**LRM_RSD**	
	**r**	**P**	**r**	**P**	**r**	**P**	**r**	**P**	**r**	**P**	**r**	**P**
SBPV_MEAN	0.106	0.753	0.13	0.61	0.043	0.928	0.065	0.928	−0.037	0.928	0.14	0.61
SBPV_SD	−0.161	0.101	0.422*	0	−0.055	0.566	0.327*	0	0.187	0.061	0.429*	0
SBPV_CV	−0.242*	0.019	0.424*	0	−0.082	0.413	0.335*	0	0.204*	0.043	0.427*	0
SBPV_ARV	−0.042	0.684	0.398*	0	−0.066	0.594	0.339*	0	0.242*	0.012	0.404*	0
SBPV_SV	−0.102	0.436	0.372*	0	−0.06	0.581	0.294*	0	0.187	0.077	0.385*	0
SBPV_RSD	−0.19	0.054	0.449*	0	−0.047	0.639	0.324*	0	0.195	0.054	0.462*	0
DBPV_MEAN	−0.026	0.816	−0.146	0.211	0.099	0.375	−0.184	0.186	−0.242*	0.032	−0.127	0.301
DBPV_SD	−0.032	0.786	0.202	0.102	−0.033	0.786	0.215	0.102	0.112	0.404	0.21	0.102
DBPV_CV	−0.06	0.615	0.257*	0.012	−0.078	0.484	0.287*	0.012	0.19	0.069	0.256*	0.012
DBPV_ARV	−0.022	0.913	0.189	0.198	−0.035	0.913	0.24	0.12	0.181	0.198	0.205	0.192
DBPV_SV	−0.003	0.989	0.151	0.487	−0.038	0.966	0.205	0.384	0.138	0.487	0.167	0.487
DBPV_RSD	−0.05	0.744	0.212	0.096	−0.029	0.769	0.202	0.108	0.108	0.386	0.224	0.096

BP blood pressure, SBP systolic BP, DBP diastolic BP, MBP mean BP, SBPV systolic blood pressure variability, DBPV diastolic blood pressure variability, SD standard deviation, CV coefficient of variation, ARV average real variability, SV successive variation, RSD residual standard deviation, LAM axial displacement in the left CCA wall, and LRM radial displacement in the left CCA wall. Significance of the estimates: *P < 0.05.

**Table 3:** Correlation between the beat-to-beat BP and right CCA indices.

	RAM_MEAN		RAM_SD		RAM_CV		RAM_ARV		RAM_SV		RAM_RSD	
	r	P	r	P	r	P	r	P	r	P	r	P
SBPV_MEAN	−0.098	0.753	0.059	0.928	0.024	0.928	−0.031	0.928	−0.001	0.987	0.032	0.928
SBPV_SD	0.088	0.383	0.225*	0.024	0.069	0.478	0.188	0.061	0.071	0.478	0.223*	0.024
SBPV_CV	0.144	0.147	0.214*	0.036	0.062	0.514	0.216*	0.036	0.081	0.413	0.224*	0.032
SBPV_ARV	0.059	0.594	0.121	0.305	0.035	0.713	0.172	0.106	0.082	0.512	0.103	0.368
SBPV_SV	0.05	0.615	0.183	0.077	0.023	0.825	0.183	0.077	0.092	0.482	0.168	0.107
SBPV_RSD	0.088	0.367	0.236*	0.02	0.09	0.367	0.193	0.054	0.082	0.385	0.235*	0.021
DBPV_MEAN	−0.151	0.203	−0.076	0.503	0.105	0.354	−0.153	0.203	−0.111	0.354	−0.105	0.354
DBPV_SD	0.023	0.786	0.221	0.102	0.055	0.736	0.138	0.386	0.059	0.735	0.19	0.12
DBPV_CV	0.055	0.622	0.271*	0.012	0.02	0.855	0.208*	0.048	0.116	0.264	0.249*	0.014
DBPV_ARV	0.022	0.913	0.08	0.704	0.008	0.929	0.142	0.256	0.074	0.708	0.065	0.718
DBPV_SV	0.004	0.989	0.111	0.515	0.001	0.989	0.111	0.515	0.052	0.867	0.088	0.664
DBPV_RSD	0.013	0.881	0.212	0.096	0.066	0.692	0.131	0.314	0.059	0.692	0.191	0.115
	**RRM_MEAN**		**RRM_SD**		**RRM_CV**		**RRM_ARV**		**RRM_SV**		**RRM_RSD**	
	**r**	**P**	**r**	**P**	**r**	**P**	**r**	**P**	**r**	**P**	**r**	**P**
SBPV_MEAN	0.131	0.61	0.14	0.61	−0.103	0.753	0.023	0.928	0.019	0.928	0.138	0.61
SBPV_SD	0.012	0.932	0.226*	0.024	−0.093	0.371	0.155	0.112	0.14	0.152	0.233*	0.024
SBPV_CV	−0.034	0.721	0.189	0.057	−0.063	0.514	0.172	0.086	0.14	0.152	0.200*	0.046
SBPV_ARV	0.042	0.684	0.312*	0	−0.059	0.594	0.253*	0.012	0.245*	0.012	0.342*	0
SBPV_SV	0.004	0.962	0.248*	0.014	−0.077	0.52	0.197	0.077	0.192	0.077	0.280*	0.006
SBPV_RSD	0.003	0.971	0.285*	0.005	−0.099	0.351	0.174	0.07	0.166	0.083	0.290*	0.005
DBPV_MEAN	0.073	0.503	−0.024	0.816	−0.075	0.503	−0.157	0.203	−0.152	0.203	−0.043	0.703
DBPV_SD	0.03	0.786	0.129	0.386	−0.031	0.786	0.12	0.386	0.096	0.453	0.13	0.386
DBPV_CV	−0.028	0.813	0.141	0.16	−0.012	0.892	0.197	0.06	0.155	0.147	0.147	0.147
DBPV_ARV	0.039	0.913	0.147	0.252	−0.025	0.913	0.168	0.2	0.151	0.252	0.174	0.2
DBPV_SV	0.056	0.867	0.103	0.55	−0.026	0.968	0.138	0.487	0.126	0.487	0.132	0.487
DBPV_RSD	0.029	0.769	0.163	0.192	−0.04	0.76	0.129	0.314	0.111	0.386	0.165	0.192

BP blood pressure, SBP systolic BP, DBP diastolic BP, MBP mean BP, SBPV systolic blood pressure variability, DBPV diastolic blood pressure variability, SD standard deviation, CV coefficient of variation, ARV average real variability, SV successive variation, RSD residual standard deviation, RAM axial displacement in the right CCA wall, and RRM radial displacement in the right CCA wall. Significance of the estimates: *P < 0.05.

**Table 4:** Canonical Correlation analysis.

	r				p
	0.787				0
BP Set Standardized Canonical Correlation Coefficients		CCA Set Standardized Canonical Correlation Coefficients			
SBPV_MEAN	−1.362	LAM_MEAN	0.089	RAM_MEAN	−0.104
SBPV_SD	4.708	LAM_SD	−0.012	RAM_SD	0.563
SBPV_CV	−4.194	LAM_CV	0.107	RAM_CV	−0.108
SBPV_ARV	0.197	LAM_ARV	−0.208	RAM_ARV	−0.601
SBPV_SV	−0.306	LAM_SV	0.294	RAM_SV	0.372
SBPV_RSD	−1.346	LAM_RSD	0.174	RAM_RSD	−0.369
DBPV_MEAN	0.865	LRM_MEAN	0.312	RRM_MEAN	−0.148
DBPV_SD	0.947	LRM_SD	0.911	RRM_SD	−0.058
DBPV_CV	2.515	LRM_CV	0.034	RRM_CV	0.093
DBPV_ARV	−0.524	LRM_ARV	0.865	RRM_ARV	−0.831
DBPV_SV	1.049	LRM_SV	−0.077	RRM_SV	0.581
DBPV_RSD	−3.269	LRM_RSD	−2.202	RRM_RSD	0.616

BP blood pressure, SBP systolic BP, DBP diastolic BP, MBP mean BP, SBPV systolic blood pressure variability, DBPV diastolic blood pressure variability, SD standard deviation, CV coefficient of variation, ARV average real variability, SV successive variation, RSD residual standard deviation, RAM axial displacement in right CCA wall, and RRM radial displacement in right CCA wall. Significance of the estimates: *P < 0.05.

**Table 5:** Canonical Correlation analysis in the EH and NEH groups.

EH group (n = 76)		NEH group (n = 62)					
r		p		r		p	
0.907		0.001		0.919		0	
Standardized Canonical Correlation Coefficients							
SBPV_MEAN	0.714	DBPV_MEAN	0.077	SBPV_MEAN	−0.89	DBPV_MEAN	0.429
SBPV_SD	−3.544	DBPV_SD	1.549	SBPV_SD	6.612	DBPV_SD	0.825
SBPV_CV	3.03	DBPV_CV	−0.641	SBPV_CV	−4.965	DBPV_CV	3.911
SBPV_ARV	−2.473	DBPV_ARV	1.022	SBPV_ARV	−0.241	DBPV_ARV	−0.002
SBPV_SV	1.722	DBPV_SV	−0.617	SBPV_SV	0.119	DBPV_SV	0.811
SBPV_RSD	−0.172	DBPV_RSD	−0.94	SBPV_RSD	−2.366	DBPV_RSD	−4.493
LAM_MEAN	0.032	LRM_MEAN	−0.012	LAM_MEAN	0.151	LRM_MEAN	0.419
LAM_SD	−0.351	LRM_SD	1.742	LAM_SD	−0.344	LRM_SD	1.207
LAM_CV	0.144	LRM_CV	0.028	LAM_CV	−0.013	LRM_CV	−0.055
LAM_ARV	−0.55	LRM_ARV	1.392	LAM_ARV	0.382	LRM_ARV	1.025
LAM_SV	0.234	LRM_SV	−0.765	LAM_SV	0.167	LRM_SV	−0.415
LAM_RSD	0.798	LRM_RSD	−2.88	LAM_RSD	−0.077	LRM_RSD	−1.958
RAM_MEAN	−0.016	RRM_MEAN	−0.2	RAM_MEAN	−0.098	RRM_MEAN	−0.13
RAM_SD	0.272	RRM_SD	−0.181	RAM_SD	0.233	RRM_SD	−0.128
RAM_CV	0.023	RRM_CV	−0.029	RAM_CV	−0.039	RRM_CV	0.261
RAM_ARV	−0.611	RRM_ARV	−0.193	RAM_ARV	−0.721	RRM_ARV	−0.632
RAM_SV	0.594	RRM_SV	−0.073	RAM_SV	0.364	RRM_SV	0.712
RAM_RSD	−0.317	RRM_RSD	0.218	RAM_RSD	0.279	RRM_RSD	−0.092

BP blood pressure, SBP systolic BP, DBP diastolic BP, MBP mean BP, SBPV systolic blood pressure variability, DBPV diastolic blood pressure variability, SD standard deviation, CV coefficient of variation, ARV average real variability, SV successive variation, RSD residual standard deviation, RAM axial displacement in the right CCA wall, and RRM radial displacement in the right CCA wall. Significance of the estimates: *P < 0.05.

**Table 6:** Canonical correlation analysis in the male and female groups.

MALE (n = 76)				FEMALE (n = 62)			
r		p		r		p	
0.919		0		0.94		0	
Standardized Canonical Correlation Coefficients							
SBPV_MEAN	0.903	DBPV_MEAN	−0.027	SBPV_MEAN	0.597	DBPV_MEAN	−0.076
SBPV_SD	−4.219	DBPV_SD	−0.831	SBPV_SD	−2.894	DBPV_SD	−0.679
SBPV_CV	4.658	DBPV_CV	−1.092	SBPV_CV	2.463	DBPV_CV	−0.352
SBPV_ARV	−2.503	DBPV_ARV	1.311	SBPV_ARV	−0.66	DBPV_ARV	−0.068
SBPV_SV	2.406	DBPV_SV	−1.781	SBPV_SV	1.244	DBPV_SV	−0.31
SBPV_RSD	−1.249	DBPV_RSD	2.766	SBPV_RSD	0.906	DBPV_RSD	0.731
LAM_MEAN	−0.047	LRM_MEAN	0.113	LAM_MEAN	0.033	LRM_MEAN	−0.418
LAM_SD	−0.097	LRM_SD	0.901	LAM_SD	−1.392	LRM_SD	−1.361
LAM_CV	0.142	LRM_CV	0.203	LAM_CV	−0.186	LRM_CV	−0.153
LAM_ARV	−1.325	LRM_ARV	0.85	LAM_ARV	−0.718	LRM_ARV	0.079
LAM_SV	0.623	LRM_SV	−0.087	LAM_SV	0.976	LRM_SV	−0.249
LAM_RSD	0.416	LRM_RSD	−2.14	LAM_RSD	1.2	LRM_RSD	1.856
RAM_MEAN	−0.03	RRM_MEAN	−0.098	RAM_MEAN	0.014	RRM_MEAN	0.105
RAM_SD	0.161	RRM_SD	−0.619	RAM_SD	0.601	RRM_SD	0.139
RAM_CV	−0.01	RRM_CV	0.038	RAM_CV	0.041	RRM_CV	0.042
RAM_ARV	−0.268	RRM_ARV	0.458	RAM_ARV	0.434	RRM_ARV	−1.173
RAM_SV	0.203	RRM_SV	−0.797	RAM_SV	−0.511	RRM_SV	0.92
RAM_RSD	0.222	RRM_RSD	0.888	RAM_RSD	−0.68	RRM_RSD	0.283

BP blood pressure, SBP systolic BP, DBP diastolic BP, MBP mean BP, SBPV systolic blood pressure variability, DBPV diastolic blood pressure variability, SD standard deviation, CV coefficient of variation, ARV average real variability, SV successive variation, RSD residual standard deviation, RAM axial displacement in the right CCA wall, and RRM radial displacement in the right CCA wall. Significance of the estimates: *P < 0.05.

**Table 7:** Canonical correlation analysis for different ages.

*Age* <= 60 (n = 72)				*Age* > 60 (n = 62)			
r		p		r		p	
0.87		0.009		0.926		0.001	
Standardized Canonical Correlation Coefficients							
SBPV_MEAN	−0.097	DBPV_MEAN	0.512	SBPV_MEAN	0.948	DBPV_MEAN	−0.418
SBPV_SD	−0.662	DBPV_SD	3.251	SBPV_SD	−4.289	DBPV_SD	−2.278
SBPV_CV	−1.073	DBPV_CV	2.194	SBPV_CV	3.326	DBPV_CV	−1.689
SBPV_ARV	−0.63	DBPV_ARV	−0.245	SBPV_ARV	−1.402	DBPV_ARV	1.427
SBPV_SV	0.192	DBPV_SV	1.379	SBPV_SV	0.912	DBPV_SV	−1.701
SBPV_RSD	0.73	DBPV_RSD	−5.378	SBPV_RSD	1.705	DBPV_RSD	4.183
LAM_MEAN	0.093	LRM_MEAN	0.026	LAM_MEAN	0.158	LRM_MEAN	−0.329
LAM_SD	0.893	LRM_SD	−0.151	LAM_SD	1.118	LRM_SD	−2.306
LAM_CV	−0.216	LRM_CV	0.208	LAM_CV	−0.144	LRM_CV	0.067
LAM_ARV	−0.503	LRM_ARV	−1.494	LAM_ARV	−0.469	LRM_ARV	−1.182
LAM_SV	0.111	LRM_SV	1.503	LAM_SV	0.077	LRM_SV	0.221
LAM_RSD	−0.664	LRM_RSD	−0.334	LAM_RSD	−0.92	LRM_RSD	3.693
RAM_MEAN	−0.104	RRM_MEAN	−0.126	RAM_MEAN	−0.078	RRM_MEAN	−0.112
RAM_SD	1.391	RRM_SD	−0.952	RAM_SD	−0.508	RRM_SD	1.208
RAM_CV	−0.404	RRM_CV	0.266	RAM_CV	−0.432	RRM_CV	0.072
RAM_ARV	−0.102	RRM_ARV	0.119	RAM_ARV	1.035	RRM_ARV	0.58
RAM_SV	0.167	RRM_SV	0.244	RAM_SV	−0.791	RRM_SV	0.357
RAM_RSD	−0.831	RRM_RSD	0.779	RAM_RSD	0.47	RRM_RSD	−1.708

BP blood pressure, SBP systolic BP, DBP diastolic BP, MBP mean BP, SBPV systolic blood pressure variability, DBPV diastolic blood pressure variability, SD standard deviation, CV coefficient of variation, ARV average real variability, SV successive variation, RSD residual standard deviation, RAM axial displacement in the right CCA wall, and RRM radial displacement in the right CCA wall. Significance of the estimates: *P < 0.05.

**Table 8:** Canonical correlation analysis for different ages (without EH).

*Age* < = 60 (n = 32)				*Age* > 60 (n = 30)			
r		p		r		p	
0.89		0.001		0.88		0	
Standardized Canonical Correlation Coefficients							
SBPV_MEAN	−0.042	DBPV_MEAN	0.074	SBPV_MEAN	0.669	DBPV_MEAN	−0.178
SBPV_SD	−0.721	DBPV_SD	1.735	SBPV_SD	−0.973	DBPV_SD	−0.636
SBPV_CV	−0.052	DBPV_CV	−4.006	SBPV_CV	1.367	DBPV_CV	−1.275
SBPV_ARV	0.391	DBPV_ARV	−0.988	SBPV_ARV	0.418	DBPV_ARV	2.509
SBPV_SV	0.225	DBPV_SV	0.298	SBPV_SV	−0.476	DBPV_SV	−1.575
SBPV_RSD	0.715	DBPV_RSD	−1.907	SBPV_RSD	0.880	DBPV_RSD	3.970
LAM_MEAN	0.002	LRM_MEAN	−0.027	LAM_MEAN	−0.003	LRM_MEAN	−0.003
LAM_SD	−0.022	LRM_SD	−0.054	LAM_SD	0.067	LRM_SD	−0.158
LAM_CV	−0.004	LRM_CV	−0.007	LAM_CV	−0.021	LRM_CV	−0.228
LAM_ARV	3.894	LRM_ARV	−7.075	LAM_ARV	−0.596	LRM_ARV	−0.334
LAM_SV	−3.842	LRM_SV	4.736	LAM_SV	0.459	LRM_SV	0.106
LAM_RSD	0.035	LRM_RSD	0.091	LAM_RSD	−0.059	LRM_RSD	1.060
RAM_MEAN	−0.002	RRM_MEAN	−0.008	RAM_MEAN	0.014	RRM_MEAN	−0.004
RAM_SD	0.014	RRM_SD	−0.239	RAM_SD	−0.010	RRM_SD	0.012
RAM_CV	0.026	RRM_CV	0.024	RAM_CV	−0.111	RRM_CV	0.019
RAM_ARV	−0.037	RRM_ARV	0.523	RAM_ARV	0.781	RRM_ARV	−1.691
RAM_SV	−0.721	RRM_SV	0.793	RAM_SV	−0.145	RRM_SV	1.036
RAM_RSD	0.036	RRM_RSD	0.347	RAM_RSD	0.022	RRM_RSD	−0.028

BP blood pressure, SBP systolic BP, DBP diastolic BP, MBP mean BP, SBPV systolic blood pressure variability, DBPV diastolic blood pressure variability, SD standard deviation, CV coefficient of variation, ARV average real variability, SV successive variation, RSD residual standard deviation, RAM axial displacement in the right CCA wall, and RRM radial displacement in the right CCA wall. Significance of the estimates: *P < 0.05.
